# Identification and Characterization of Poorly Differentiated Invasive Carcinomas in a Mouse Model of Pancreatic Neuroendocrine Tumorigenesis

**DOI:** 10.1371/journal.pone.0064472

**Published:** 2013-05-14

**Authors:** Karen E. Hunter, Marsha L. Quick, Anguraj Sadanandam, Douglas Hanahan, Johanna A. Joyce

**Affiliations:** 1 Cancer Biology and Genetics Program, Memorial Sloan-Kettering Cancer Center, New York, New York, United States of America; 2 Louis V. Gerstner Jr. Graduate School of Biomedical Sciences, Memorial Sloan-Kettering Cancer Center, New York, New York, United States of America; 3 Swiss Institute of Bioinformatics (SIB), Lausanne, Switzerland; 4 Swiss Institute for Experimental Cancer Research (ISREC), School of Life Sciences, Swiss Federal Institute of Technology Lausanne (EPFL), Lausanne, Switzerland; Stanford University, United States of America

## Abstract

Pancreatic neuroendocrine tumors (PanNETs) are a relatively rare but clinically challenging tumor type. In particular, high grade, poorly-differentiated PanNETs have the worst patient prognosis, and the underlying mechanisms of disease are poorly understood. In this study we have identified and characterized a previously undescribed class of poorly differentiated PanNETs in the RIP1-Tag2 mouse model. We found that while the majority of tumors in the RIP1-Tag2 model are well-differentiated insulinomas, a subset of tumors had lost multiple markers of beta-cell differentiation and were highly invasive, leading us to term them poorly differentiated invasive carcinomas (PDICs). In addition, we found that these tumors exhibited a high mitotic index, resembling poorly differentiated (PD)-PanNETs in human patients. Interestingly, we identified expression of Id1, an inhibitor of DNA binding gene, and a regulator of differentiation, specifically in PDIC tumor cells by histological analysis. The identification of PDICs in this mouse model provides a unique opportunity to study the pathology and molecular characteristics of PD-PanNETs.

## Introduction

Pancreatic neuroendocrine tumors (PanNETs) are a rare but clinically challenging tumor type; a consequence of marked disease heterogeneity and limited understanding of the molecular basis for these cancers, among other factors. PanNETs arise from cells of the neuroendocrine system within the pancreas and include insulinomas, gastrinomas, glucagonomas, VIPomas and somatostatinomas [1]. Well-differentiated, low to medium grade PanNETs can be classified into two groups: functional tumors that secrete hormone, which represent 30% of patients, and nonfunctional tumors which do not secrete hormone [2]. Well-differentiated PanNETs are clinically distinct from poorly differentiated, high-grade PanNETs (PD-PanNETs), which are characterized by a high mitotic index [3].

PanNETs are the second most common pancreatic neoplasms, representing approximately 1.3% of pancreatic cancers in incidence and 10% of cases in prevalence [4]. PanNETs have diverse clinical outcomes, in which some patients can exhibit long-term survival, although the overall 10-year survival rate is only 40%. PanNET patients with nonfunctioning tumors constitute a disproportionate number of patients with poor prognosis, as they grow silently and present with extensive metastatic disease at diagnosis. Patients with PD-PanNETs represent the worst prognosis of the entire PanNET spectrum [5].

It is currently unknown whether nonfunctioning tumors and PD-PanNETs arise from a different cell of origin to hormone-producing neoplasms, or reflect a more stem-like differentiation status [1]. While it is generally accepted that well-differentiated PanNETs arise from the various neuroendocrine cells of the pancreas, the cell of origin for poorly differentiated PanNETs is controversial. It has been proposed that PD-PanNETs may in fact originate from a separate, potentially non-neuroendocrine lineage [3]. Therefore, insights into PanNET tumorigenesis from animal models may be informative in discriminating between these different possibilities.

The RIP1-Tag2 (RT2) mouse model of islet-cell carcinoma has proven very instructive in studying neuroendocrine tumor progression, and in particular, in predicting clinical efficacy of new therapeutics [6]. In this model, beta-cell specific expression of the SV40 T-antigen leads to islet-cell carcinomas through a reproducible and well-characterized tumor progression pathway [7]. The RT2 mouse model utilizes a viral oncogene, SV40 T-antigen, to inactivate the p53 and retinoblastoma (Rb) tumor suppressor pathways and induce tumorigenesis in pancreatic islet cells. While this is not the mechanism of tumor initiation in humans, a recent study has shown that negative regulators of the p53 pathway are aberrantly activated in approximately 70% of PanNETs [8]. Increased levels of these negative regulator proteins, MDM2, MDM4 and WIP1, thus leads indirectly to a decrease in p53 activity [8]. In addition, another recent study showed that 68% of PanNET tumor samples examined exhibited attenuation of the Rb pathway, via increased CDK4/6 [9]. Therefore, the RT2 model inactivates the same tumor suppressor pathways as genetic alterations observed in a majority of PanNET patients.

Several different classes of tumor grade have been reported in the RT2 model including encapsulated tumors, microinvasive and invasive carcinomas, which are thought to represent a multi-stage progression series [10], [11]. In addition, a previous study has suggested that a subset of tumors may arise through a separate pathway [12]. Through miRNA profiling of a panel of tumors and metastases, they identified a subset of tumors whose gene expression signature clusters closely with that of liver metastases, and termed these “met-like primary” (MLP) tumors. It was proposed that these MLPs most likely occur through a divergent branch during tumorigenesis [12], separate from the prototypical progression from encapsulated to invasive classes of tumors.

In this study, we have identified and characterized a previously undescribed class of poorly differentiated PanNETs in the RT2 model. We found that while the majority of tumors in the RT2 model are well-differentiated insulinomas, a subset of tumors were found to be poorly differentiated and highly invasive, leading us to term them poorly differentiated invasive carcinomas (PDICs). These tumors had lost expression of multiple markers of beta-cell differentiation. In addition, we found that these tumors exhibited a high mitotic index, resembling PD-PanNETs in human patients. We also identified the inhibitor of differentiation/inhibitor of DNA binding gene Id1 as being specifically expressed by these tumors by histological analysis, and also to be expressed in “met-like primary” tumors. The identification of PDICs provides a unique opportunity to study the pathology and molecular characteristics of PD-PanNETs in an experimental model.

## Results

### Identification of a novel class of invasive, poorly differentiated tumors

**Figure 1:**
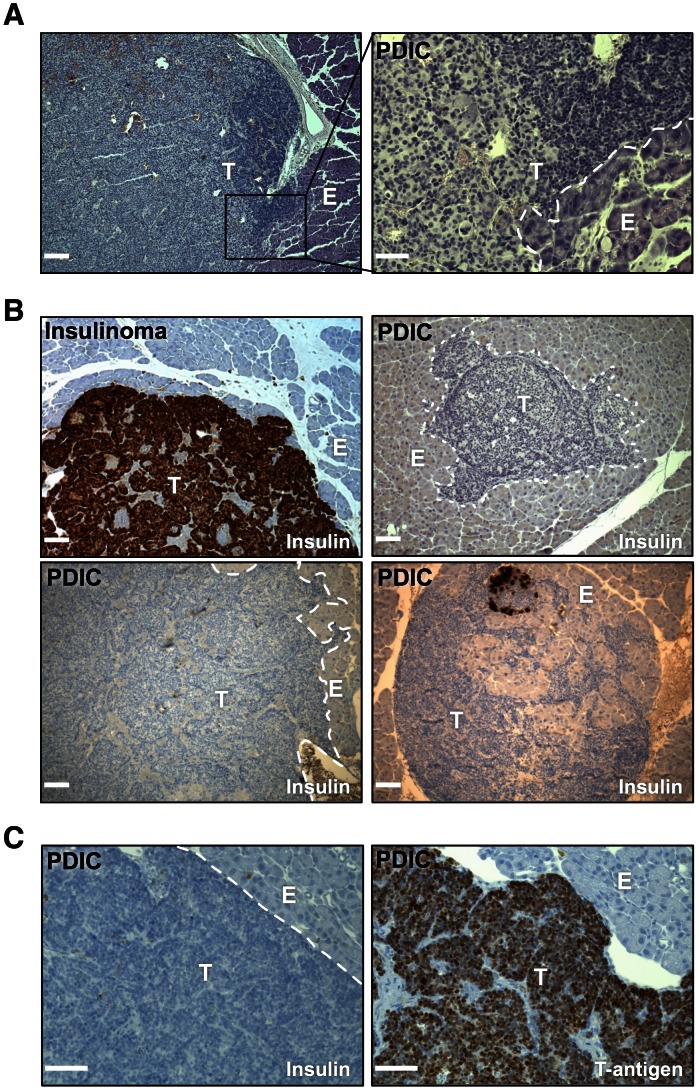
Identification of poorly differentiated invasive carcinomas (PDICs). (A) H&E staining of paraffin tissue sections demonstrates a tumor region with an anaplastic appearance and a high nuclear to cytoplasm ratio. 40× magnification of the boxed region is shown in the right panel. T =  Tumor, E =  Exocrine pancreas. Scale bars: 100 μm (left), 20 μm (right). (B) IHC for insulin was performed on paraffin sections from RT2 mice, and representative images are shown. While the majority of the tumors (labeled insulinoma) produce high levels of insulin (detected by DAB in brown), poorly differentiated invasive carcinomas (PDICs) are negative for insulin staining and are highly invasive. Scale bar: 100 μm. (C) Adjacent sections were stained for insulin and T-antigen. PDICs remained positive for T-antigen staining. Scale bar: 50 μm.

During the course of analysis of serially sectioned pancreata from RT2 mice, we identified a previously undescribed class of tumors. These tumors were found histologically due to their high nuclear to cytoplasmic ratio, anaplastic morphology and extensive invasion into the surrounding normal exocrine pancreas (Figure 1A). While the majority of insulinoma tumors in RT2 mice express high levels of insulin, these highly invasive tumors were found to lack insulin expression, consistent with their poorly differentiated appearance (Figure 1B). These tumors appeared to be a distinct class of tumors from the invasive classes previously described in this model [10], leading us to term these tumors poorly differentiated invasive carcinomas (PDICs).

Discovery of a set of tumors that had lost insulin expression was surprising, as it had been previously thought that all RT2 tumors expressed insulin at high levels due to their origin from beta-cells. By H&E staining the PDICs appeared to be PanNETs, however there remained the possibility that these arose from a different cell of origin and were not PanNETs. To determine whether they were still driven by the SV40 T-antigen oncogene, which induces tumorigenesis in beta-cells under control of the rat insulin promoter (RIP) [7], we stained sections with a T-antigen antibody, and found that all tumors expressed T-antigen, including all PDICs that had lost insulin expression (Figure 1C).

### PDICs exhibit a high mitotic index

**Figure 2:**
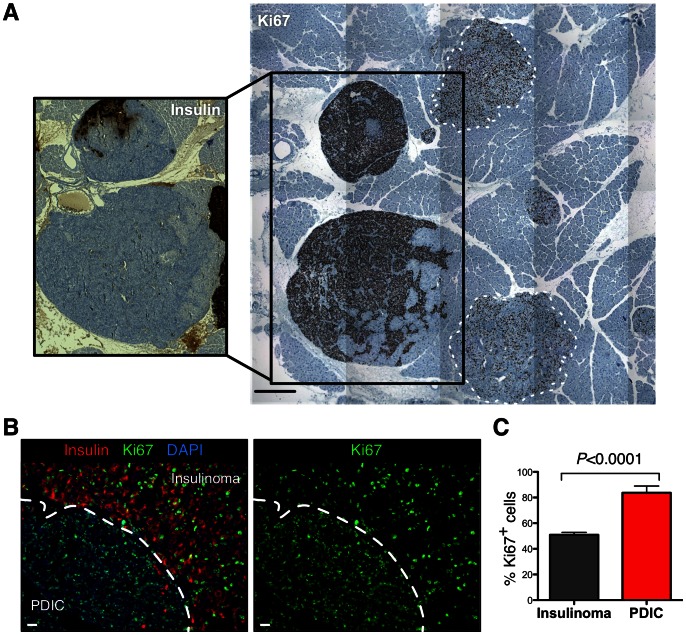
PDICs exhibit a high mitotic index. (A) Adjacent tumor sections were stained for insulin and Ki67. PDICs that do not express insulin (inset) were found to have a very large proportion of Ki67^+^ proliferating cells. Tumors outlined with dotted white lines on the right are insulinomas, showing a markedly lower degree of Ki67 staining. Scale bar represents 200 μm. (B) Tumor sections were stained by immunofluorescence for insulin and Ki67. Insulinomas (top right) exhibit significantly less Ki67 positive cells than adjacent PDICs (lower left). Scale bar: 20 μm. (C) Mitotic index was calculated by the number of Ki67^+^ cells over the total cells per tumor, and tumors were stratified into insulinomas or PDICs using insulin staining. *P* values were obtained using Student's unpaired t-test.

High-grade PanNETs in patients are characterized by their high mitotic index [3]. Therefore, we were interested in whether these PDICs also exhibited an increased mitotic index. As shown in Figure 2A, PDICs that have lost insulin (shown in the inset), have a markedly higher proportion of cells that are Ki67 positive, as compared to tumors that maintain insulin expression, shown on the right side of the image. Co-staining of insulin and Ki67 confirmed that PDICs which have lost insulin exhibit a higher mitotic index compared to tumors that maintain insulin expression (Figure 2B). We quantified the mitotic index in PDICs compared to insulinomas and found that PDICs exhibited a significantly higher mitotic index, with greater than 80% of all cells being Ki67^+^ (Figure 2C). Therefore, PDICs in the RT2 model are highly proliferative, similar to poorly differentiated, high-grade PanNET tumors in patients.

### PDICs occur in the majority of RT2 mice

PDICs have not been previously described in the RT2 model and it was unknown whether these tumors were rare or present in all mice but had been previously missed. To thoroughly investigate the frequency of PDICs, we completely sectioned through the entire pancreas of RT2 mice and examined every 10^th^ slide to ensure that each tumor throughout the tissue was represented and analyzed. When staining for insulin, we found that while all mice had seven to ten insulinomas each, 70% of WT RT2 mice examined also had at least one PDIC. Thus, our analysis has revealed that PDICs are actually not uncommon, constituting 10.3% of all tumors (Table 1).

**Table 1:** Frequency of poorly differentiated invasive carcinomas (PDICs) in RT2 mice.

	RT2 Mice	RT2 Tumors
**Total Analyzed**	10	86
**PDIC**	7	9
**Percentage PDIC**	**70%**	**10.3%**

RT2 pancreata were serially sectioned and every 10^th^ slide was stained for insulin to identify PDICs, determined by loss of insulin expression. PDICs were confirmed by expression of Id1. RT2 mice have multiple tumors, thus the frequency of PDIC incidence per mouse and as a percentage of all tumors was calculated (70% and 10.3% respectively).

### PDICs exhibit loss of neuroendocrine markers

**Figure 3:**
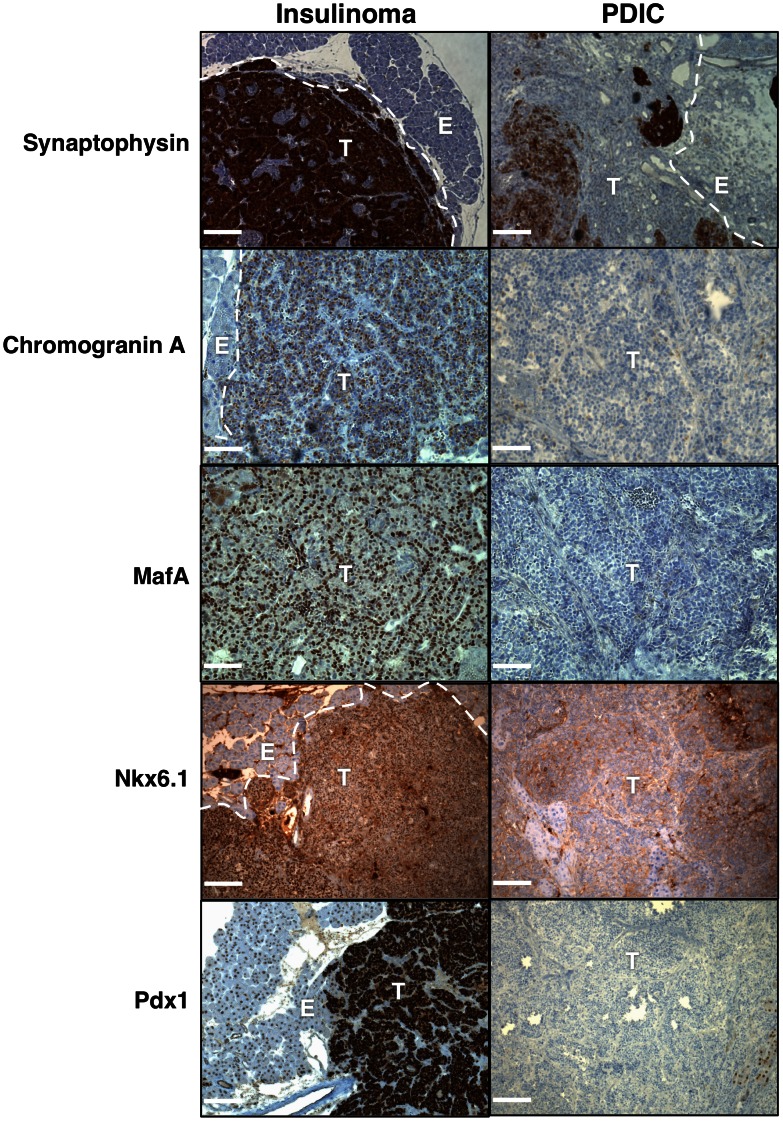
PDICs exhibit loss of multiple neuroendocrine markers. Tissue sections were stained for the following markers of neuroendocrine differentiation: Synaptophysin, ChromograninA, MafA, Nkx6.1 and Pdx1, and representative images are shown. While insulinomas all stained positive for these markers (left panels, detected by DAB in brown), PDICs exhibited either complete or heterogeneous loss of these markers (right panels). T =  tumor, E =  exocrine pancreas. Scale bars: 50 μm.

Given the loss of insulin expression in PDICs, we next investigated whether other markers of beta-cells and neuroendocrine cells were also absent in these tumors. Tissue sections containing both PDICs and insulinomas were stained for a panel of cell type-specific markers [13], [14]. Synaptophysin, a marker of neuroendocrine cells, was found to be generally expressed in both insulinomas and PDICs, indicating that these tumors still maintained aspects of neuroendocrine differentiation, however some PDICs exhibited heterogeneous downregulation (Figure 3). An additional neuroendocrine marker, chromogranin A, was found to be completely absent in PDICs. Moreover, the transcription factors MafA, Nkx6.1 and Pdx1 were also absent in PDICs (Figure 3). Therefore, while PDICs maintain some aspects of neuroendocrine cell differentiation, they exhibit loss of multiple markers of β-cell differentiation.

**Figure 4:**
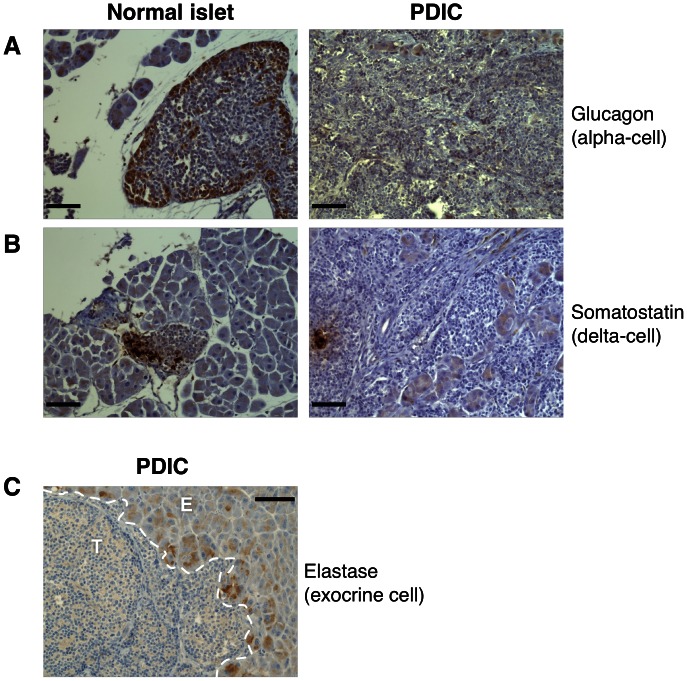
PDICs do not express alpha-cell, delta-cell or exocrine cell markers. (A, B) Tissues were stained for glucagon, to label alpha-cells, and somatostatin, to label delta-cells. Normal islets showed the expected expression pattern for these markers at the periphery of islets (detected by DAB in brown). PDICs were negative for these pancreatic endocrine cell markers, exhibiting staining in rare alpha-cells or delta-cells seen within the tumor and with non-specific background staining in exocrine cells. (C) Tissues were stained for elastase, which labels acinar cells in the exocrine pancreas. Positively stained cells are labeled in brown, and were only observed in the exocrine tissue (E), never in the PDIC tumors (T). Scale bar: 50 μm.

As PDICs have lost expression of beta-cell specific genes, it raised the possibility that they are tumors that have arisen from one of the other neuroendocrine cell types that constitute pancreatic islets. While beta-cells represent the major cell type in islets, alpha-cells and delta-cells are also present, and tumors such as glucagonomas and somatostatomas can arise from these cells [1]. Also, it has previously been shown that under extreme beta-cell loss, alpha-cells can be converted into beta-cells [15], indicating that there can be plasticity between the different neuroendocrine cell types of the islets. Therefore, it is also possible that these tumors could reflect the converse situation, in which beta-tumor cells trans-differentiate into one of the other pancreatic neuroendocrine cell types. To address this possibility, we stained tissue sections for glucagon, a marker of alpha-cells, and somatostatin, a marker of delta-cells. We saw that normal islets exhibited a small proportion of cells that stained positive for these cell markers, as expected. Alpha- and delta-cells were also observed in tumors, albeit at a lower frequency, as they were displaced by hyperproliferation of the tumor cells (Figure 4A, B). However, we saw that there was no glucagon or somatostatin staining of tumor cells in PDICs (Figure 4A, B), indicating that PDICs lack expression of all pancreatic neuroendocrine cell type-specific markers. Finally, as it has been suggested that pancreatic acinar cells may serve as progenitor cells for pancreatic islets [16], [17], we investigated whether PDICs showed any evidence of exocrine cell expression. We examined expression of elastase, a digestive enzyme produced by the acinar cells [18] (Figure 4C). Exocrine cells surrounding PDICs stained positively for elastase, as expected. We found no evidence for elastase staining in tumor cells of PDICs, indicating these tumors do not arise from an exocrine cell of origin.

### PDICs specifically express Id1

Characterization of PDICs revealed that they have lost multiple markers of beta-cell differentiation, however, factors that were specifically expressed by these tumors remained unknown. We therefore undertook a candidate-based approach to determine a specific, positive marker of PDICs. Due to the apparent dedifferentiation of these tumors, we investigated the expression pattern of Id1, one of the inhibitor of DNA binding (Id) proteins. Id1 has been shown to inhibit differentiation, to stimulate proliferation and to be expressed by embryonic stem cells, adult stem cells and cancer stem cells [19]–[21], thus making it a logical candidate to investigate.

**Figure 5:**
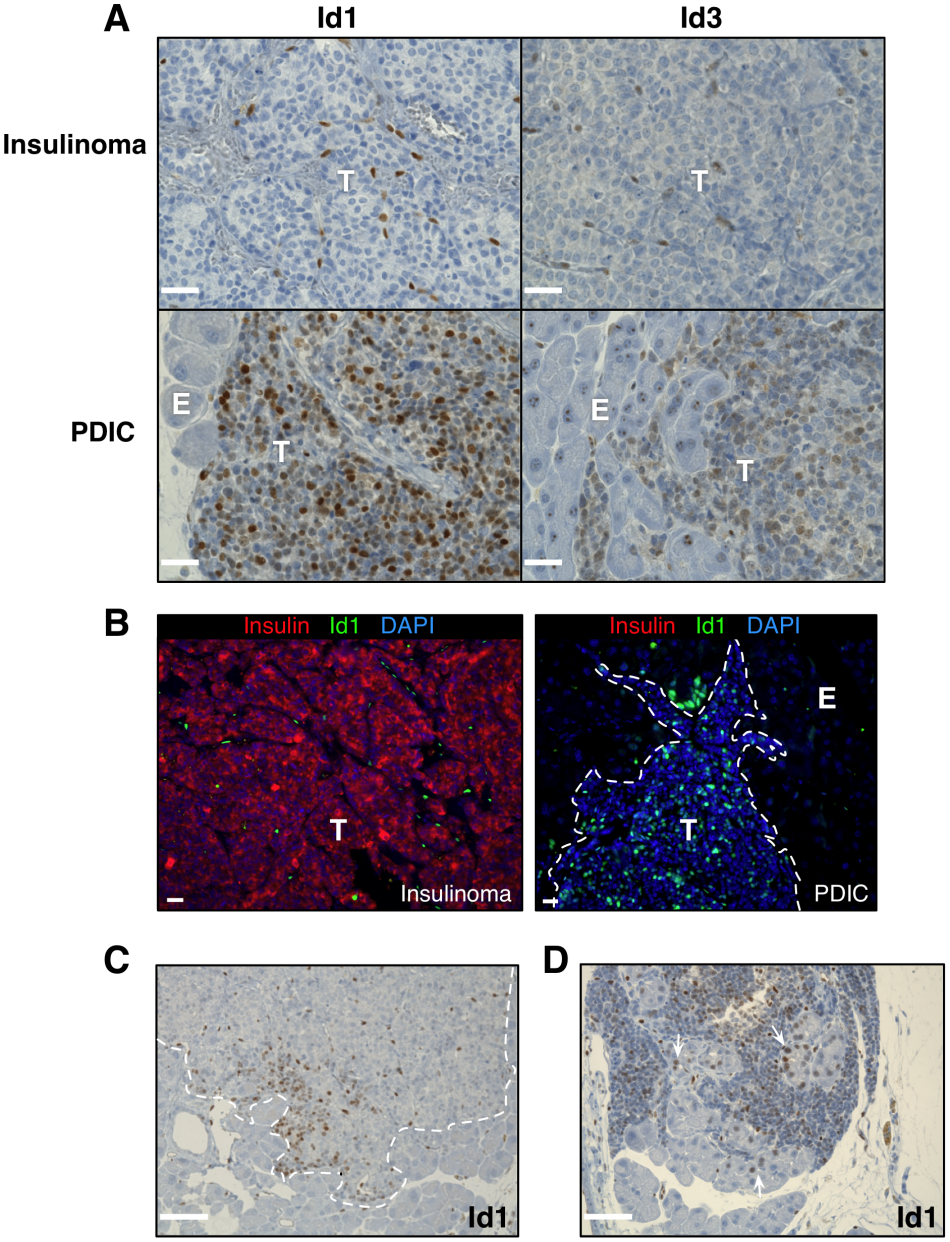
Id1 is expressed by tumor cells in PDICs. (A) Paraffin sections were stained for Id1 and Id3 and representative images are shown. PDIC tumor cells specifically expressed Id1 and Id3, while insulinoma tumor cells did not, with Id1 and Id3 staining only detectable in endothelial cells as expected. Scale bar: 50 μm. (B) Id1 and insulin expression are mutually exclusive by immunofluorescence staining. Scale bar: 20 μm. (C) Id1^+^ cells (brown) are evident at the invasive front of a tumor, and (D) Id1 staining is observed in exocrine cells adjacent to a PDIC (indicated by white arrows). Scale bar: 50 μm.

We stained tissue sections from RT2 mice and found that Id1 was expressed in all endothelial cells within tumors (Figure 5A), which was expected as Id1 has been shown to play an important role in tumor angiogenesis [22]. Tumor cells in insulinomas had no detectable Id1 staining (Figure 5A). Strikingly, we found that the majority of tumor cells in PDICs specifically stained for Id1, exhibiting a nuclear staining pattern (Figure 5A). We also found that tumor cells in PDICs specifically were positive for Id3 (Figure 5A), another Id family member which can play a redundant role to Id1 [19]. By staining each of the PDICs that had been previously identified by their loss of insulin, we determined that tumor cells in all PDICs were positive for Id1. In addition, through immunofluorescence co-staining we found that Id1 expression was mutually exclusive from insulin expression (Figure 5B). While the majority of PDIC tumors were found to be entirely positive for Id1, we found two instances in which Id1 was regionally expressed; for example, present only in tumor cells along the invasive front of the PDIC, with the remainder of the tumor only having Id1 expression in endothelial cells (Figure 5C). Interestingly, we also observed several tumors in which Id1 nuclear staining was found in the exocrine cells directly adjacent to a PDIC (Figure 5D), while we never saw exocrine Id1 staining in any other instance.

**Figure 6:**
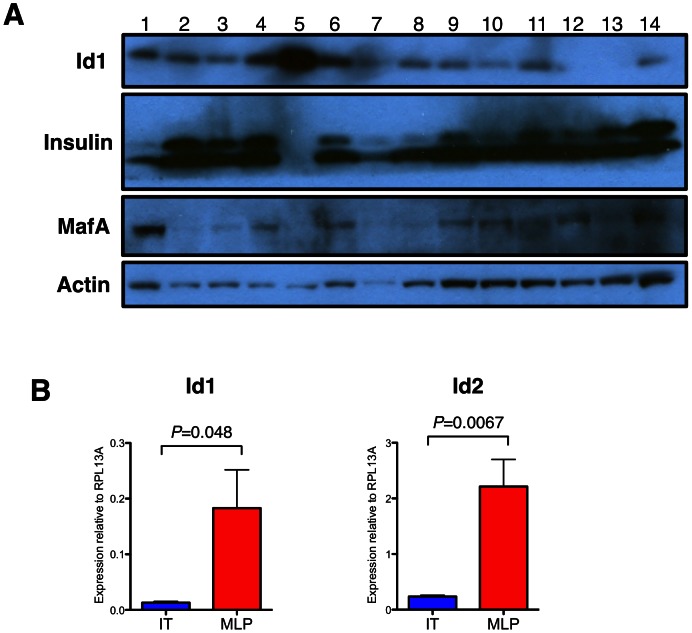
PDICs and “met-like primary” tumors have high expression of Id1. (A) A panel of protein lysates from individual RT2 tumors was blotted for Id1, insulin and MafA, with actin as a loading control. The tumor lysate #5 is a PDIC, as it has high Id1 levels with absence of insulin and MafA protein expression. (B) Expression of Id1 and Id2 were examined in RNA from invasive tumors (IT) and “met-like primary” tumors (MLP) from the RT2 model, and calculated compared to the housekeeping gene RPL13A. Both Id1 and Id2 are expressed at significantly higher levels in MLP compared to IT tumors. *n* = 4 tumors for each group. *P* values were obtained using Student's unpaired t-test.

Additionally, we confirmed the expression of Id1 in protein lysates from a panel of RT2 tumors (Figure 6A). Most tumors showed moderate levels of Id1, most likely originating from the Id1 produced by endothelial cells within the vasculature of the tumor. Interestingly, one tumor showed much higher levels of Id1 (Figure 6A, lane #5). Consistent with the immunohistochemistry staining of PDICs (Figure 3), we found that this tumor had corresponding absences of insulin and MafA expression (Figure 6A).

A previous study has suggested that in the RT2 model, a subset of tumors may arise through a distinct pathway, termed “met-like primary” (MLP) tumors [12]. It was proposed that these MLPs most likely occur through a divergent branch during tumorigenesis, separate from the multistage progression from encapsulated to invasive classes of tumors. The relatively low frequency of MLPs, also constituting ∼10% of the tumors profiled [12], and showing a distinct gene signature from the majority of the tumors, raised the interesting possibility that met-like primary tumors are in fact PDICs.

To investigate whether MLPs exhibited similar markers to PDICs, we analyzed the expression of specific Id family members in RNA in tumors that had previously been identified as MLPs based on a previously reported gene signature (Olson et al., 2009). We found that expression of Id1 and Id2 were significantly upregulated in MLPs, as compared to invasive tumors (IT) (Figure 6B). These results suggest that MLPs and PDICs may indeed be the same type of tumor, potentially representing a common and separate pathway for these more aggressive tumors compared to other classes of tumors in this model.

## Discussion

### Identification of PDICs in RT2 mice

We have described the discovery of a class of tumors, PDICs, which have lost markers of beta-cell differentiation, are highly proliferative and anaplastic. Intriguingly, this tumor type exhibits many of the characteristics of high grade PD-PanNETs, a tumor subset of which very little is known and for which patients have very poor prognosis. Studying these tumors may thus provide important insights into the molecular mechanisms of PD-PanNETs.

Having serendipitously discovered these tumors through their loss of insulin expression, we first further characterized these tumors histologically. We found that PDICs had not only lost insulin expression, but did not express many markers of beta-cell differentiation, including those that are expressed in progenitor cells during development. *MafA* is a transcription factor responsible for insulin activation and is expressed only in mature beta-cells [14]; thus its absence in RT2 PDICs was consistent with the loss of insulin. Two other transcription factors were found to be absent: *Nkx6.1* and *Pdx1*. *Nkx6.1* is turned on in endocrine cell progenitors, remains expressed during differentiation and is important for endocrine differentiation [14]. *Pdx1* has been shown to be essential for pancreatic development and beta-cell maturation and is expressed in pancreatic progenitors and in immature and mature beta-cells [14]. Interestingly, synaptophysin, which is expressed by many cells of the neuroendocrine and neural lineage [13], maintains at least some expression in PDICs, suggesting that there is still maintenance of some aspects of their neuroendocrine specification.

We also excluded the possibility that these tumors were derived from, or trans-differentiated into, a different cell type within the pancreas by staining for markers of other pancreatic neuroendocrine cells and acinar cells. PDICs did not express markers of alpha-cells or delta-cells, and maintained expression of T-antigen. It is intriguing that tumors that have lost insulin expression still maintain T-antigen expression, as its expression is driven by the rat insulin promoter (RIP), which is controlled in a similar manner to that of the endogenous mouse insulin gene. The mechanism of this silencing of insulin expression remains an open question.

The inhibitor of DNA binding family member Id1 was the first protein that we identified as being specifically expressed in PDICs. Id1 has been shown to be expressed by adult neural stem cells and glioma stem cells [20], [21]. This, combined with the loss of markers of endocrine progenitors, raises the interesting possibility that these tumors may have stem cell-like properties, or perhaps have arisen from a pancreatic progenitor cell, however these possibilities remain to be investigated.

### How do PDICs develop?

Having identified and characterized PDICs histologically, we were interested in understanding how they develop. There are several hypotheses as to how these tumors may arise. PDICs could result from a further progression from the invasive IC2 class of carcinomas, in which loss of differentiation markers has occurred during the progression to a high-grade tumor. Alternatively, PDICs could represent a separate tumorigenesis pathway, in which they progress without beta-cell markers in a distinct development pathway to insulinomas. Similarly, PDICs could represent a completely different tumor type, derived from a different cell of origin, whether it be a stem or progenitor cell or another pancreatic neuroendocrine cell.

We have made several interesting observations that could provide insight into these possibilities regarding the development of PDICs. The majority of PDICs were found to be entirely Id1^+^, suggesting that tumors arose from a single clone, consistent with the hypothesis that these tumors originated through a separate pathway or cell of origin to the other classes of RT2 tumor. However, we found that occasional tumors exhibited Id1^+^ cells only at the invasive edges of the tumor. This could be indicative of the progression of an invasive tumor cell population that has lost insulin expression, and gained Id1 expression. Alternatively, it could again represent a subset of tumor cells that have arisen from a PDIC clone, which is present at the tumor edge. The observation that PDICs exhibit a high mitotic index also brings into question the timing of their development. With high proliferation rates, it would be expected that PDICs would grow at a much faster rate than insulinomas. However, PDICs are not any larger on average than insulinomas, suggesting that they may arise later than insulinomas. Thus, the development of these tumors remains an interesting, open question and could provide important insights into the development of patient PD-PanNETs.

Due to the similarly low ∼10% frequency of PDICs found in our study and the previously described met-like primary (MLP) tumors [12], we were intrigued as to whether these tumors were the same. Interestingly, we found that *Id1* and *Id2* are expressed highly in MLP RT2 tumors, suggesting that PDICs may indeed represent the same lesion as MLPs. It has been previously proposed that MLPs develop through a distinct development pathway to other invasive tumors [12]. This is consistent with our observation that the majority of PDICs are entirely insulin negative, and thus unlikely to have progressively ‘lost’ insulin expression during the course of multistage tumorigenesis. It will be interesting in future studies to investigate an extensive panel of endocrine markers in MLPs, including insulin and several of the beta-cell specific genes analyzed here, to definitively determine if MLPs and PDICs are the same class of tumor.

### Implications for PD-PanNET patients

Surgical resection is the most effective treatment option for PanNETs; however, approximately 65% of patients present with unresectable or metastatic disease. PD-PanNETs respond very differently to therapeutic agents than well-differentiated PanNETs and thus are treated with a different regimen. PD-PanNETs are generally managed with surgery and platinum-based chemotherapy [23]. Unfortunately, little is currently known about how to stratify patients based on their prognosis, nor the use of targeted therapies for PD-PanNETs with unresectable metastases. The RT2 model has been shown to have critical predictive value for clinical studies, predicting response to the recently FDA-approved drugs sunitinib malate and everolimus for well-differentiated PanNETs [24]–[27]. Previously, studies in the RT2 model have been used to investigate the behavior of well-differentiated PanNETs. Our identification of PDICs in this model that mimic many characteristics of PD-PanNETs in patients now offers a unique opportunity to explore the pathology and molecular characteristics of this aggressive tumor type.

## Materials and Methods

### Transgenic mice and tissue processing

The generation and characterization of RIP1-Tag2 (RT2) [7] mice has been previously reported. All animal studies were performed using protocols approved by the Animal Care Committee at Memorial Sloan-Kettering Cancer Center. RT2 mice were sacrificed by heart perfusion with PBS followed by 10% zinc-buffered formalin. Tumor-containing pancreas and control tissues were removed, formalin-fixed overnight, processed through an ethanol series, embedded in paraffin blocks and paraffin sections (5 μm) were cut on a microtome.

### Immunohistochemistry (IHC) and immunofluorescence (IF) staining

Paraffin sections were stained using DAB detection with a Discovery XT automated staining processor (Ventana Medical Systems, Inc). For immunofluorescence staining, paraffin sections were processed with a Discovery XT automated staining processor, incubated with the primary antibody of interest overnight at 4°C, incubated with the corresponding fluorescently-tagged secondary antibody for 1 hour at room temperature, incubated with 46-diamidino-2-phenyl indole (DAPI) for 10 minutes and mounted with ProLong Gold (Invitrogen). Tissue sections were visualized under a Carl Zeiss Axioimager Z1 microscope and images were acquired with Axiovision using an Apotome (Zeiss) or with TissueFAXS (TissueGnostics, Vienna, Austria). For the proliferation analysis, the quantitation was performed using HistoQuest software (TissueGnostics) to determine the percentage of Ki67^+^ cells. The following antibodies were used for IF and IHC staining: rabbit anti-SV40 T antigen (Santa Cruz, 1∶500), guinea pig anti-insulin (DAKO, 1∶1000), rabbit anti-synaptophysin (DAKO, 1∶200), rabbit anti-Ki67 (Vector, 1∶200), rabbit anti-chromogranin A (Abcam 1∶250), rabbit anti-MafA (Abcam, 1∶500), anti-Nkx6.1, rabbit anti-Pdx1 (Abcam, 1∶500), rabbit anti-glucagon (Millipore 1∶1000), rabbit anti-somatostatin (DAKO, 1∶500), rabbit anti-elastase (Abcam, 1∶2000), rabbit anti Id1 (BioCheck, 1∶200) and rabbit anti-Id3 (BioCheck, 1∶200). Relevant species-specific IgG controls for each antibody were stained in parallel, and no non-specific staining was observed.

### Western blotting

Protein lysates were made from dissected RT2 tumors using RIPA lysis buffer. 40 μg of protein was loaded onto SDS-PAGE gels and transferred to PVDF membranes for immunoblotting. Membranes were probed with antibodies against Id1 (BioCheck 1∶500), insulin (DAKO 1∶1000), MafA (Abcam 1∶1000) and actin (Sigma, 1∶5000) and detected using HRP-conjugated anti-guinea pig or anti-rabbit (Jackson Immunoresearch) antibodies using chemiluminescence detection (Amersham).

### Gene expression analysis in RT2 tumors

RNA was isolated from different tumors (invasive tumors (IT) and “met-like primary” tumors (MLPs)) from the RT2 mouse model and reverse transcription was performed as described [12]. Quantitative real-time RT-PCR was performed using Rotor-Gene (Qiagen) as per the instructions from the manufacturer. ΔΔ(cycle threshold)ct values were calculated after normalizing the ct values for each individual gene to that of the housekeeping gene, RPL13A, as described [28].

### Statistical analysis

Throughout this study, means +/− SEM (standard error of the mean) are reported unless otherwise specified. For all two-way comparisons, unpaired t-tests were used and were considered statistically significant if *P*<0.05.
